# H6N6 Avian Influenza Virus Infection Induced Pyroptosis of M1 Macrophages by Activating Caspase-1

**DOI:** 10.3390/v17111492

**Published:** 2025-11-12

**Authors:** Hui Zhu, Dongfang He, Sicong Liu, Xiaohui Fan, Lingxi Gao, Liping Guo, Zengfeng Zhang

**Affiliations:** 1 Department of Microbiology, Guangxi Medical University, Nanning 530021, China; zhuhui3546@126.com (H.Z.);; 2 Key Laboratory of Basic Research on Regional Diseases (Guangxi Medical University), Education Department of Guangxi Zhuang Autonomous Region, Nanning 530021, China

**Keywords:** avian influenza virus (AIV), H6N6 subtype, M1 macrophages, caspase-1, pyroptosis

## Abstract

The H6N6 avian influenza virus has expanded its host range from birds to mammals. Some strains can now bind to human-like receptors, raising concerns about human infection. Although H6N6 is a low-pathogenic avian influenza virus (LPAIV), it is unclear whether it triggers pyroptosis in human lungs, a process linked to cytokine storms in infections like H7N9. Here, we found that the chicken-origin H6N6 LPAIV can effectively replicate in and infect human alveolar macrophages and their M1 macrophages. Viral infection of M1 macrophages upregulated the mRNA levels of NLRP3, caspase-1, and Gasdermin D (GSDMD). Subsequently, caspase-1 was activated and cleaved GSDMD protein into its N-terminal fragment (GSDMD-N), which formed pores in the cell membrane and triggered the release of IL-1β and IL-18. Further analysis demonstrated that inhibition of the NLRP3/Caspase-1/GSDMD pathway by specific inhibitors attenuated pyroptosis in infected M1 macrophages. In summary, our study revealed that H6N6 virus infection induces M1 macrophage pyroptosis via the NLRP3/caspase-1/GSDMD pathway. Notably, M1 macrophages inherently produce pro-inflammatory cytokines; their pyroptosis, accompanied by the release of IL-1β and IL-18, can amplify inflammation and potentially trigger a cytokine storm in the lungs. These findings reveal novel pathogenic mechanisms and potential therapeutic targets for avian influenza viruses.

## 1. Introduction

In August 2024, the World Health Organization (WHO) released an updated list of over 30 dangerous viral and bacterial pathogens. These pathogens, including the influenza A virus, could potentially trigger the next pandemic [1]. Over the past two decades, avian influenza viruses (AIVs) such as H5N1, H7N9, and H5N6 have repeatedly crossed inter-species barriers from birds to infect humans, posing a serious threat to public health [2,3,4]. Moreover, it is concerning that the H6 subtype of AIV is widely prevalent among wild waterfowl, domestic waterfowl, and terrestrial birds on the Eurasian continent. While its human receptor-binding affinity (SAα-2,6 Gal) appears weaker than pandemic strains like H7N9, emerging evidence suggests certain H6N6 strains have acquired sufficient binding capacity to initiate human infection [5,6]. When avian influenza viruses infect humans, their primary target cells are type II alveolar epithelial cells and alveolar macrophages in the lower respiratory tract, leading to alveolar tissue damage and even severe pulmonary pathology [7,8]. Alveolar macrophages, the predominant resident innate immune cells in lung tissue, are highly heterogeneous and plastic. They can polarize into M1 or M2 phenotypes in response to local microenvironments [9]. M1 macrophages, primarily activated by lipopolysaccharides (LPS) and IFN-γ, secrete pro-inflammatory cytokines and exhibit antimicrobial functions. M2 macrophages are mainly activated by IL-4, IL-13 and macrophage colony-stimulating factor (MCSF), and they secrete anti-inflammatory cytokines, exhibiting potent anti-inflammatory activity and promoting tissue repair [10]. However, an excessive inflammatory response caused by M1 macrophages—referred to as a “cytokine storm”—can lead to fatal outcomes [11,12].

PANoptosis is an inflammatory programmed cell death that includes apoptosis, pyroptosis, and necroptosis [13]. Among them, pyroptosis is triggered by inflammatory caspases and is characterized by cell swelling and osmotic lysis, which result in cytomembrane rupture and the release of pro-inflammatory factors (IL-1β, IL-18) [13,14,15]. Pyroptosis can be divided into the canonical pathway, the non-canonical pathway, the caspase-3/8–mediated pathway, and the granzyme-mediated pathway [15,16,17,18,19,20,21]. The canonical pathway involves pathogen-associated molecular patterns (PAMPs) activating the NLRP3 inflammasome, which cleaves pro-caspase-1 to generate active caspase-1; this activated caspase then cleaves Gasdermin D (GSDMD) to form membrane pores [14]. In non-canonical pyroptosis, human caspase-4/5 (caspase-11 in mice) is activated by the direct binding of LPS to their N-terminal caspase activation and recruitment domain (CARD). The activated caspase-4/5/11 then cleaves GSDMD [16,17,18]. Pyroptosis is a crucial component of the body’s innate immune response and plays a key role in antiviral infection. Specifically, moderate pyroptosis benefits the host by inhibiting viral replication and spread. However, excessive pyroptosis can induce autoinflammatory and autoimmune diseases. Although pyroptosis causes cell death, it also triggers inflammatory reactions by inducing the release of IL-1β and IL-18. This recruits more inflammatory cells, expands inflammation, and can lead to immune damage [22,23]. Influenza A virus infection can trigger programmed cell death (PCD), including apoptosis, necroptosis, and pyroptosis [24]. Moreover, this virus is a common viral activator of NLRP3 inflammasome and often induces pyroptosis. It has been confirmed that pyroptosis causes the cytokine storm and acute lung injury induced by highly pathogenic avian influenza virus H7N9 infection [23,25]. Serological epidemiological surveys have detected H6 antibodies in the serum of workers exposed to the virus in China, indicating an occupational exposure risk [26]. Although no human infections with H6N6 virus have been reported to date, experiments confirm that H6N6 replicates efficiently in human alveolar epithelial cells and macrophages in vitro [27]. However, whether low-pathogenic avian influenza virus (LPAIV) H6N6 infection induces pyroptosis and its pathway remains unclear.

Given that M1 macrophages play a pivotal role in initiating inflammatory responses during viral infections, and considering their well-documented capacity to produce pro-inflammatory cytokines that can contribute to cytokine storms, we specifically investigated the interaction between chicken-origin H6N6 AIV and human M1 macrophages. This focus was further justified by our preliminary findings showing H6N6’s efficient replication in both human alveolar macrophages and their M1-polarized counterparts. Our study had two primary objectives: (1) to determine whether H6N6 infection induces pyroptosis in M1 macrophages, and (2) to elucidate the role of this programmed cell death in host antiviral responses and viral pathogenesis. We found that chicken-origin H6N6 AIV could efficiently replicate and infect human alveolar macrophages and M1 macrophages. H6N6 virus infection induces pyroptosis of M1 macrophages via the NLRP3/caspase-1/GSDMD pathway. Inhibiting this pathway with specific inhibitors attenuated pyroptosis in infected cells. It is noteworthy that M1 macrophages inherently produce pro-inflammatory cytokines, and simultaneous pyroptosis with release of IL-1β and IL-18 could further increase pro-inflammatory cytokine levels, potentially leading to a cytokine storm in the lungs. These results provide new insights into the pathogenic mechanisms of H6N6 avian influenza virus and identify potential therapeutic targets.

## 2. Materials and Methods

### 2.1. Virus and Cell Lines

The H6N6 subtype avian influenza virus strain A/CK/DG/2953/2021 (DG2953) was isolated using 9- to 11-day-old embryonated chicken eggs. It was provided by Prof. Yi Guan from the Joint Institute of Virology (Shantou University–the University of Hong Kong). Viral stocks were prepared in Madin-Darby Canine Kidney (MDCK) cells and titrated using hemagglutination assay and 50% tissue culture infectious dose (TCID_50_) methods. The human acute monocytic leukemia cell line THP-1 was obtained from the cell bank of the Chinese Academy of Sciences.

### 2.2. H6N6 Avian Influenza Virus Replication in Human Lung Tissue

Human lung tissue specimens were obtained from surgical resections provided by the First Affiliated Hospital of Guangxi Medical University. Specifically, the tissue used in this study was obtained from a 40-year-old male donor who had undergone surgical resection for lung cancer. The samples were strictly collected from non-tumor regions that were histopathologically confirmed as normal within the resected lung tissue. To control for donor variability, we implemented standardized protocols for tissue processing (uniform transport, digestion, and infection conditions), included reference virus controls in each experiment, and validated through statistical analysis of historical data showing no significant donor-dependent effects on viral replication. Suspected cancerous and/or other abnormal tissues were removed from the specimens before cutting the normal lung tissue into 0.2 cm × 0.2 cm × 0.2 cm blocks. Two blocks of human lung tissue were first selected to test for influenza virus infection. One block was ground to obtain the supernatant for virus isolation and culture. The other was used to detect viral antigens to ensure the specimens were not infected with influenza virus. Next, lung tissue blocks were placed into a 6-well cell culture plate and rinsed with F-12K tissue culture medium containing penicillin (Solarbio, Beijing, China) and streptomycin (Solarbio, Beijing, China). They were then inoculated with 1 × 10^6^ TCID_50_ of virus in a volume of 500 μL medium and cultured at 37 °C and 5% CO_2_ (99.5% purity, Nanning Lantian Medical Gas Co., Nanning, China) for 1 h. PBS was used as a mock control. Afterwards, the tissues were rinsed and incubated with F-12K medium (Gibco, New York, NY, USA) containing 0.2% TPCK-trypsin (Sigma-Aldrich, St. Louis, MO, USA) and 1% bovine serum albumin (BSA) (Gibco, New York, NY, USA). Tissues were collected at 12, 24, and 48 h post-infection (hpi), then fixed in 4% (*v*/*v*) formaldehyde (Biosharp, Hefei, China) in PBS at room temperature for 24 h. The viral antigen was detected by immunohistochemistry (IHC) method. After antigen retrieval, the primary antibody against nucleoprotein (NP) (kindly provided by the National Institute of Diagnostics and Vaccine Development in Infectious Diseases, Xiamen University) (1:500) was added and incubated overnight at 4 °C. Subsequently, goat anti-mouse IgG-specific biotin conjugate (Calbiochem, Darmstadt, Germany) (1:50) was added, followed by hematoxylin (Sigma-Aldrich, St. Louis, MO, USA) counterstaining. The types of cells infected by the virus in alveolar tissue, especially alveolar macrophages, were microscopically observed.

### 2.3. The THP-1 Cells’ Polarization into M1 Macrophages

THP-1 cells were cultured in R/MINI-1640 medium (Gibco, New York, NY, USA) containing 10% fetal bovine serum (Gibco, New York, NY, USA), 100 U/mL penicillin, and 100 μg/mL streptomycin. THP-1 cells were seeded at 2.0 × 10^6^ cells/well in a 6-well plate. Then, 100 nmol/L phorbol 12-myristate 13-acetate (PMA) (Sigma-Aldrich, St. Louis, MO, USA) was added to each well. The plate was placed in a 37 °C, 5% CO_2_ incubator for 48 h to stimulate THP-1 cells to differentiate into M0 macrophages. The medium with M0 induction solution was removed, and the cells were washed with PBS. Then, M1 induction solution containing 10 ng/mL LPS (Sigma-Aldrich, St. Louis, MO, USA) and 20 ng/mL IFN-γ (Sigma-Aldrich, St. Louis, MO, USA) was added. The cells were incubated at 37 °C in 5% CO_2_ to polarize into M1 macrophages. RNA was extracted from M1 macrophages lysates after virus infection and lysis. RNA was extracted using the RNAiso Plus kit (Takara, Osaka, Japan), and cDNA was synthesized via reverse transcription with the PrimerScript™ RT Reagent Kit with gDNA Eraser (Takara, Osaka, Japan). Quantitative real-time PCR (qRT-PCR) was then used to detect the mRNA expression of M1 macrophage-related marker genes, including CXCL-10, TNF-α, IL-6, and COX-2. This analysis verified the polarization of THP-1 cells into M1 macrophages.

### 2.4. CCK8 Detection of M1 Macrophage Viability

M1 macrophages were suspended and seeded at 6 × 10^4^ cells per well into 96-well plates. Cells were infected with 100 μL/well of H6N6 virus at multiplicities of infection (MOI) of 0.001, 0.01, or 0.1, with different wells receiving different MOIs, and experiments were conducted in triplicate for each different MOI. Plates were incubated at 37 °C under 5% CO_2_ for 1 h. After removing the virus solution, 100 μL of DMEM (Gibco, New York, NY, USA) supplemented with 2% FBS was added to each well, and plates were returned to a 37 °C, 5% CO_2_ incubator for continued culture. At 12, 24, and 48 hpi, 10 μL of CCK-8 (Biosharp, Hefei, China) was added per well and incubated at 37 °C in a 5% CO_2_ incubator for 1 h. Optical density (OD) was measured at 450 nm using a microplate reader. Cell viability (%) = (OD value of virus − OD value of blank)/(OD value of mock − OD value of blank) × 100%.

### 2.5. Lactate Dehydrogenase (LDH) Release Assay

When the cell membrane is damaged, LDH, a soluble enzyme present in all living cells, is released into the surrounding extracellular space. Therefore, the presence of LDH in the culture medium is used as a marker for cell apoptosis [28]. M1 macrophages were suspended and seeded into 24-well plates at 2 × 10^5^ cells per well. Then, 1 mL/well of virus dilution (MOI = 0.1) was added, and the cells were incubated at 37 °C with 5% CO_2_ for 1 h. After removing the virus inoculum, the wells were washed twice with PBS and subsequently replaced with 500 μL/well of virus culture medium. Plates were further incubated until harvest at 12, and 24 hpi. Supernatants were transferred to 1.5 mL microcentrifuge tubes and centrifuged at 1500 rpm for 5 min to remove debris. Then, 120 μL of clarified supernatant was transferred to a 96-well plate, and 60 μL/well of LDH detection reagent (Beyotime Biotechnology, Shanghai, China) was added. Plates were incubated for 30 min at room temperature in the dark. The OD was measured at 492 nm using a microplate reader. Concurrently, cells treated with lysis buffer from the LDH assay kit were used as the maximum enzyme release control. LDH release rate was calculated as follows: LDH release (%) = [(OD of virus-infected sample − OD of mock sample)/(OD of maximum enzyme release control − OD of mock sample)] × 100%.

### 2.6. Scanning Electron Microscopy (SEM) for Cell Morphology

Glass coverslips were placed in 6-well plates, ensuring full contact with the well surface. M1 macrophages were suspended and seeded at 2 × 10^6^ cells per well. Virus dilutions (MOI = 0.1) prepared in serum-free RPMI 1640 (Gibco, New York, NY, USA) were added at 2 mL per well. The plates were incubated at 37 °C with 5% CO_2_ for 1 h. After incubation, the viral solution was removed, and the wells were gently washed with PBS. Then, Virus medium (2 mL per well) was added for continued incubation. Subsequently, the medium was removed, and the wells were washed twice with PBS. At 12 and 24 h, the virus growth medium was removed, and the plates were washed with PBS and dried. Then, 2 mL of electron microscope fixative (Servicebio, Wuhan, China) was added to each well, followed by dehydration, drying, and conductivity treatment. Finally, the cytological morphology was observed using a scanning electron microscope, and images were collected.

### 2.7. Detection of Genes and Proteins in the Caspase-1-Dependent Pyroptosis Pathway

RNA and total cellular protein were extracted from M1 macrophages lysates after virus infection and lysis. RNA was extracted using the RNAiso Plus kit (Takara, Osaka, Japan), and cDNA was synthesized via reverse transcription with the PrimerScript™ RT Reagent Kit with gDNA Eraser (Takara, Osaka, Japan). RT-qPCR was then used to detect the mRNA expression of NLRP3, Caspase-1, and GSDMD. Cell proteins were lysed with RIPA lysis solution (Beyotime Biotechnology, Shanghai, China). Western blot (WB) was performed to detect the protein expression of full-length GSDMD and its cleaved N-terminal fragment (GSDMD-N). The primary antibodies of GSDMD (Cat #97558), GSDMD-N (Cat #97558), and β-actin (Cat #4970S) were obtained from Cell Signaling Technology (MA, USA); the secondary antibody (Cat #FDR007) was obtained from Fude Biological Technology Co., LTD. Furthermore, β-actin was used as the internal control for the Western blot (WB) analysis. Supernatants from M1 macrophage cultures were harvested and assayed by ELISA for inflammatory cytokines IL-1β and IL-18 using ELISA Kit (Elabscience, Wuhan, China).

The inhibitor pretreatment conditions for M1 macrophages were rigorously optimized through comprehensive dose–response experiments, ultimately establishing the following protocol: Cells were pretreated with 10 μM MCC950 (an NLRP3-specific inhibitor), 10 μM AC-YVAD-CMK (a caspase-1-specific inhibitor), and 10 μM LDC7559 (a GSDMD-specific inhibitor) for 1 h prior to H6N6 virus inoculation. All inhibitors were procured from MCE (Monmouth Junction, NJ, USA), with the selected concentrations demonstrating >75% target inhibition efficiency while maintaining >85% cell viability in validation assays. To capture the dynamic progression of H6N6-induced pyroptosis, we initially conducted a time-course experiment assessing key markers (LDH release, GSDMD cleavage, IL-1β secretion) at 12, 24, and 48 hpi. At the 48 hpi, extensive lytic cell death resulted in significant cell detachment and monolayer disintegration, precluding reliable protein quantification. Furthermore, LDH assay signals reached saturation, compromising data reproducibility. Consequently, the 12- and 24-hpi were selected for all subsequent experiments, as they reliably capture the initiation and peak phases of the pyroptosis cascade while preserving sample integrity for accurate biochemical analysis.

### 2.8. Statistical Analysis

Statistical analysis was performed using SPSS 26.0 statistical software. The experimental data were quantitative data. If they follow a normal distribution and have equal variances, a two independent samples *t*-test was used. If the experimental data have unequal variances, non-parametric tests were used for statistical analysis. The data results are presented as mean ± standard deviation (mean ± SD), the test level α = 0.05, *p* < 0.05 indicates a statistically significant difference (*p* < 0.05).

## 3. Results

### 3.1. H6N6 Subtype Avian Influenza Virus Effectively Replicates in Human Lung Tissue

**Figure 1:**
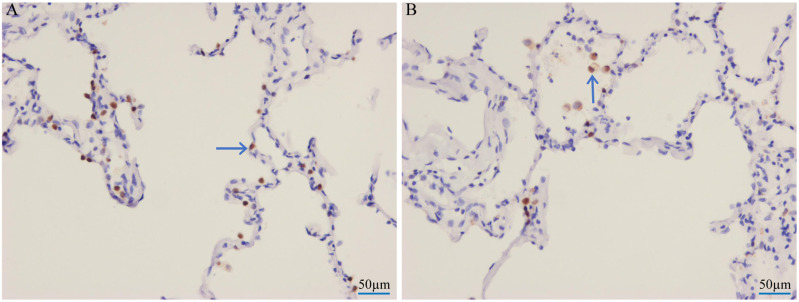
The H6N6 virus replication in human lungs inoculated with H6N6 viruses in vitro. Immunohistochemical method was used to detect the virus NP protein in lungs inoculated with the A/CK/DG/2953/2021 (H6N6). In figure (**A**), the arrow denotes alveolar cells, whereas in figure (**B**), it marks macrophages (cell identity validated by CD68, a specific macrophage marker, in prior double immunofluorescence experiments). Scale bars, 50 μm.

Human lung tissue explants were collected at 12, 24, 36, and 48 hpi with the H6N6 subtype avian influenza virus. Subsequently, the viral NP in the lung tissue was detected using IHC. To specifically identify alveolar macrophages among infected cells, we referenced our prior validation using CD68 (a canonical macrophage marker)—in early exploratory experiments, double immunofluorescence staining (CD68 for macrophages and NP for viral infection) confirmed that cells with NP signals localized to alveolar spaces (Figure 1B) were alveolar macrophages. Consistent with this, IHC results here showed NP expression in two key cell populations: alveolar cells (Figure 1A) and alveolar macrophages (Figure 1B). This indicates that the chicken-origin H6N6 virus can effectively replicate in and infect human lung tissue without prior adaptation, especially alveolar macrophages. This result suggests that the H6N6 avian influenza virus has potential for human infection.

### 3.2. The THP-1 Cells Successfully Polarized into M1 Macrophages

**Figure 2:**
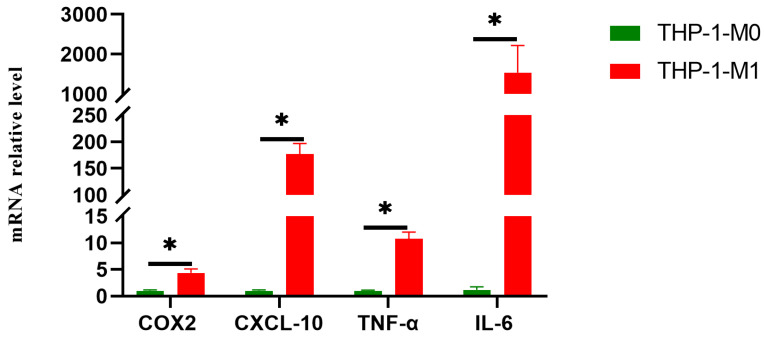
Determination of M1 polarized macrophages. RT-qPCR was used to measure the mRNA expression levels of M1 macrophage-related marker genes (CXCL-10, TNF-α, COX2 and IL-6). Note: THP-1-M0: M0 macrophages; THP-1-M1: M1 macrophages. Asterisks indicate the statistical significance as follows: *, *p* < 0.05.

THP-1 cells were first differentiated into M0 macrophages using PMA. Subsequently, these M0 macrophages were polarized into M1 macrophages by treatment with LPS and IFN-γ. RT-qPCR was used to quantify the mRNA expression levels of M1-associated markers (CXCL-10, TNF-α, COX-2, and IL-6). M1-polarized cells exhibited significantly upregulated expression of all four markers compared to M0 macrophages (*p* < 0.001) (Figure 2). These results confirm M1 macrophage polarization and establish a validated cellular model for subsequent experiments.

### 3.3. H6N6 AIV Infection Induces Cytotoxicity and Pyroptotic Morphology in M1 Macrophages

**Figure 3:**
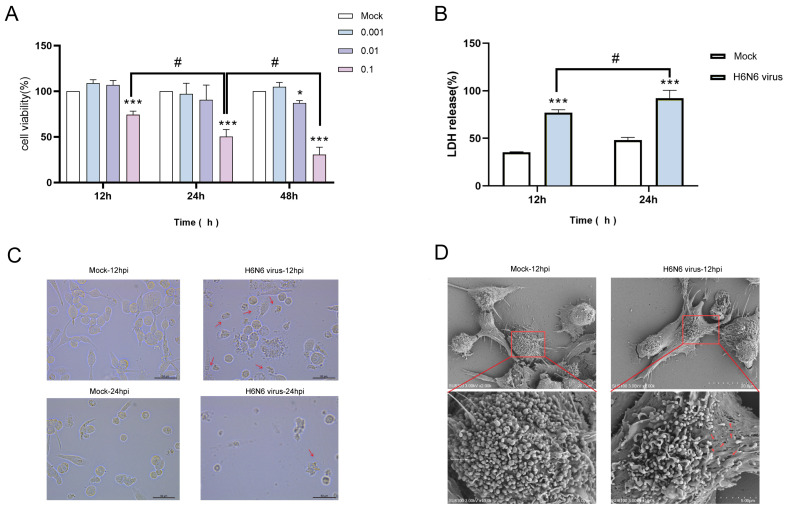
H6N6 Avian Influenza Virus Triggers Caspase-1-Dependent Pyroptosis in M1 Macrophages. (**A**) Effect of H6N6 virus infection of M1 macrophages on cell viability. (**B**) LDH releases of H6N6 virus infection of M1 macrophages. LDH releases of H6N6 virus infection of M1 macrophages was measured with optical density. (**C**) Results from optical microscopy at 12 h post-inoculation with H6N6 virus showed cytopathic effects including cell rounding, increased cytoplasmic granularity, cell necrosis, lysis, and shedding. (**D**) Scanning electron microscopy images of at 12 h post inoculated with H6N6 virus. The lower panel shows a higher magnification view of the area outlined by the red rectangle in the upper panel. The red arrows indicating the perforations on the cell membranes. Asterisks indicate the statistical significance as follows: *, *p* < 0.05 compared to the mock group; ***, *p* < 0.001 compared to the mock group. #, indicates *p* < 0.05 for the time points of viral action on cells when MOI is 0.1.

We assessed the impact of H6N6 virus at different MOIs on M1 macrophage viability using the CCK-8 assay. At 12, 24, and 48 hpi with a MOI of 0.1, H6N6-infected M1 macrophages showed significantly reduced viability compared to mock controls at all time points (*p* < 0.001). Moreover, viability decreased in a time-dependent manner with prolonged infection (*p* < 0.05). Additionally, a time-dependent decrease in viability was observed with prolonged infection (*p* < 0.05) (Figure 3A). These results demonstrate that H6N6 virus suppresses M1 macrophage viability. Based on the CCK-8 data, a MOI of 0.1 and time points of 12, and 24 hpi were selected as the optimal parameters for subsequent experiments.

LDH release serves as a validated biomarker for pyroptosis, reflecting plasma membrane damage and lytic cell death. Following infection of M1 macrophages with H6N6 virus, LDH levels in culture supernatants were quantified at 12, and 24 hpi. Compared to the mock group, the H6N6 virus-infected group showed significantly elevated LDH release at both time points (*p* < 0.001). Moreover, a time-dependent increase in LDH release was observed with prolonged infection (*p* < 0.05) (Figure 3B). Because pyroptosis causes plasma membrane rupture, these data indicate that H6N6 infection triggers pyroptosis in M1 macrophages.

Results from optical microscopy at 12 h and 24 h post-inoculation with H6N6 virus showed cytopathic effects including cell rounding, increased cytoplasmic granularity, cell necrosis, lysis, and shedding (Figure 3C). Pyroptosis is characterized by plasma membrane pore formation and cellular swelling. SEM was performed to visualize morphological changes in M1 macrophages infected with H6N6 virus (MOI 0.1) at 12, and 24 hpi. Compared to mock controls, which displayed intact, smooth plasma membranes, infected cells exhibited significant cellular swelling, reflecting osmotic imbalance, and numerous pore-like structures dispersed across the plasma membrane surface. These pores, indicated by red arrows in Figure 3D, varied in size and were frequently observed in areas of membrane blebbing and disruption, consistent with the expected morphology of GSDMD-mediated pore formation. These morphological alterations are hallmark indicators of M1 macrophages infected with the H6N6 virus and undergoing pyroptotic cell death.

### 3.4. H6N6 Avian Influenza Virus Triggers Caspase-1-Dependent Pyroptosis in M1 Macrophages

**Figure 4:**
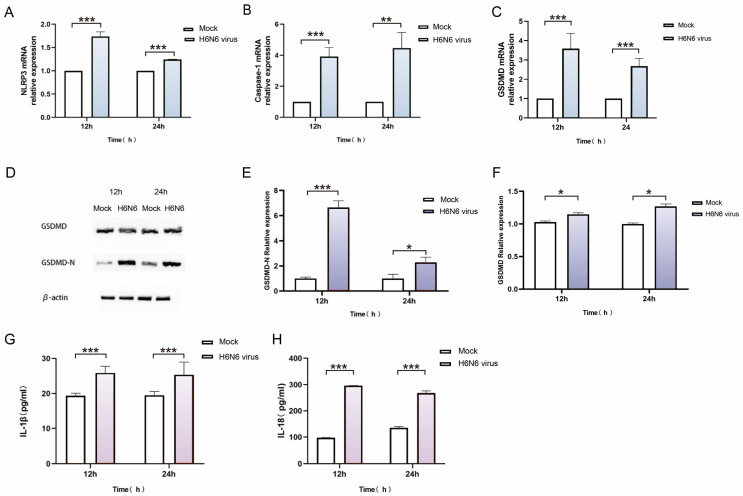
Determination of pyroptosis-related factors in M1 macrophages infected with H6N6 virus. (**A**–**C**) Determination of the mRNA expression of NLRP3, Caspase-1 and GSDMD in M1 macrophages infected with H6N6 virus with RT-qPCR. (**D**) Western blot assay was used for detecting the expressions GSDMD and GSDMD-N proteins. (**E**,**F**) Quantification of the GSDMD and GSDMD-N proteins based on the Western blot assay. (**G**,**H**) ELISA for detection of the IL-1β and IL-18 protein. Asterisks indicate the statistical significance as follows: *, *p* < 0.05; **, *p* < 0.01; ***, *p* < 0.001.

To investigate whether H6N6 virus infection triggers pyroptosis in M1 macrophages, qRT-PCR was performed to detect mRNA expression of key pyroptosis genes (NLRP3, caspase-1, and GSDMD). Compared to mock controls, H6N6 infection significantly upregulated NLRP3, caspase-1, and GSDMD mRNA at 12, and 24 hpi (*p* < 0.05) (Figure 4A–C). WB analysis revealed that the protein expression of both GSDMD and its cleaved form, GSDMD-N, was markedly increased in infected cells (*p* < 0.01). For the quantification of these Western blot band intensities (Figure 4E,F), ImageJ software (Version 1.53m) was employed. The gray value of each target band (GSDMD, GSDMD-N) was measured and then normalized to the internal control β-actin through division of the gray value of the target protein band by that of the corresponding β-actin band to obtain the relative expression level. Each experiment was repeated three times (Figure 4D–F). This suggests that the accumulation of GSDMD-N is not merely a result of proportional degradation of full-length GSDMD. Instead, mechanisms such as enhanced cleavage efficiency of GSDMD or increased stability of GSDMD-N likely contribute to this phenomenon, though further experiments are needed to confirm these hypotheses. Upregulation of NLRP3, CASP1, and GSDMD-N are core components of the caspase-1-dependent canonical pyroptosis pathway. These results suggest that H6N6 virus infection activates this pathway. Accumulation of cleaved GSDMD-N is the hallmark execution event in pyroptosis. GSDMD-N oligomerizes to form plasma membrane pores, which induce osmotic lysis, cellular swelling, and inflammatory cell death. Detection of cleaved GSDMD-N protein provided direct biochemical evidence of pyroptosis. When cells undergo pyroptosis through activation of caspase-1, activated caspase-1 cleaves pro-IL-18 and pro-IL-1β into IL-18 and IL-1β. These cytokines are then released extracellularly through pores formed by GSDMD-N in the plasma membrane. Thus, the release of IL-1β and IL-18 serves as a functional indicator of pyroptosis. Supernatants from H6N6-infected M1 macrophages were collected at 12, and 24 hpi time points. ELISA results showed a significant increase in IL-1β and IL-18 release in H6N6-infected groups (*p* < 0.001) (Figure 4G,H). This demonstrates that H6N6 infection induces pyroptosis in M1 macrophages, resulting in increased release of inflammatory cytokines IL-1β and IL-18. Taken together, these findings demonstrate that H6N6 infection induces pyroptosis in M1 macrophages via activation of caspase-1.

### 3.5. Pharmacological Inhibition Confirms the Essential Role of NLRP3/Caspase-1/GSDMD Pathway in H6N6-Induced Pyroptosis

**Figure 5:**
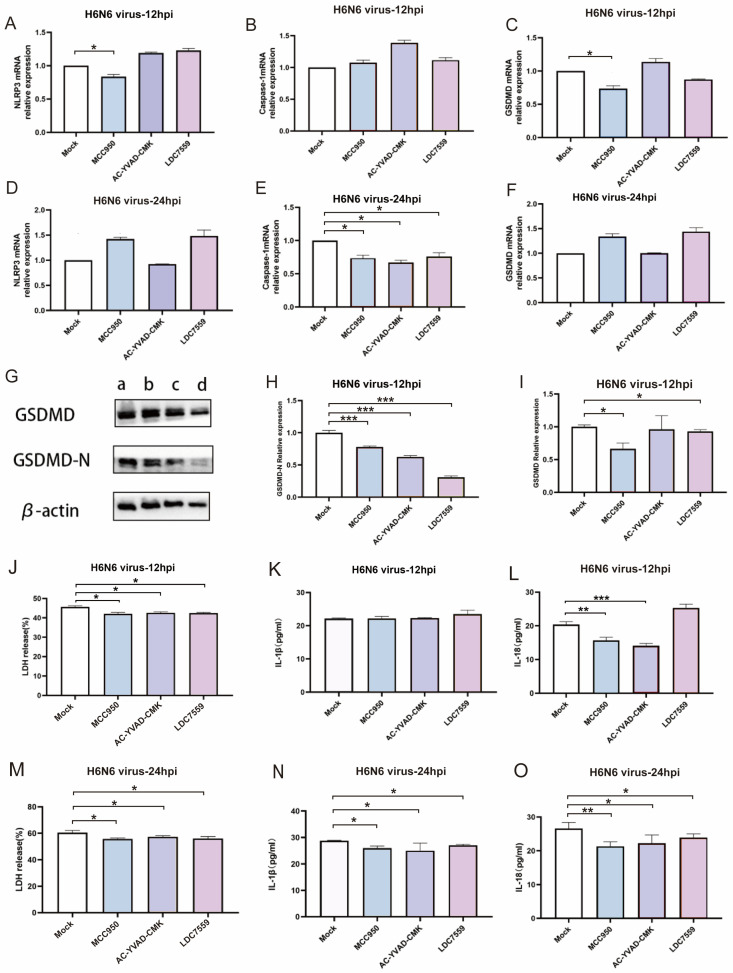
Determination of H6N6 avian influenza virus induces M1 macrophage pyroptosis via caspase-1-dependent pathway. M1 macrophages were pre-treated with MCC950 (NLRP3-specific inhibitor), AC-YVAD-CMK (Caspase-1-specific inhibitor) and LDC7559 (GSDMD-specific inhibitor) and then inoculated with the H6N6 virus. (**A**–**F**) Determination of the mRNA expression of NLRP3, caspase-1 and GSDMD at 12, 24 hpi in M1 macrophages with RT-qPCR. (**G**) Western blot assay was used for detecting the expressions GSDMD and GSDMD-N proteins, the a, b, c, and d in the WB band chart represent the mock group, MCC950 group, AC-YVMD-CMK group, and LDC7559 group, respectively. (**H**,**I**) Quantification of the GSDMD and GSDMD-N proteins based on the Western blot assay. (**J**,**M**) LDH releases of M1 macrophages was measured with optical density. ELISA for detection of the IL-1β (**K**,**N**) and IL-18 (**L**,**O**) protein. Asterisks indicate the statistical significance as follows: *, *p* < 0.05; **, *p* < 0.01; ***, *p* < 0.001.

The above experimental results suggest that the H6N6 virus infection induces pyroptosis in M1 macrophages via the canonical caspase-1-dependent pathway. However, the mechanism underlying how the virus induces pyroptosis remains relatively complex and requires further investigation, involving multiple signaling pathways and regulatory factors. To conclusively demonstrate that H6N6 induces pyroptosis via the NLRP3/Caspase-1/GSDMD pathway, we first pretreated M1 macrophages with specific inhibitors: MCC950 for NLRP3, AC-YVAD-CMK for Caspase-1, and LDC7559 for GSDMD. Then, we infected these inhibitor-treated M1 macrophages with the H6N6 virus. Finally, we detected the mRNA levels of NLRP3, caspase-1, and GSDMD, the protein level of GSDMD-N, and the release of LDH, IL-1β, and IL-18. Here, MCC950 (NLRP3 inhibitor) pre-treatment significantly downregulated NLRP3 and GSDMD mRNA expression in M1 macrophages inoculated with H6N6 virus at 12 h (*p* < 0.05). Additionally, all inhibitors (MCC950, AC-YVAD-CMK, LDC7559) markedly suppressed caspase-1 mRNA expression at 24 hpi (*p* < 0.05) (Figure 5A–F). Therefore, to some degree, the inhibitor induces downregulation of pyroptosis core genes (NLRP3, caspase-1, GSDMD), which demonstrates at the transcriptional level that H6N6 infection triggers M1 macrophage pyroptosis via the caspase-1 pathway. GSDMD cleavage to produce GSDMD-N is the essential execution step in pyroptosis. GSDMD-N oligomerizes to form plasma membrane pores that induce lytic death, a process shared by both canonical (caspase-1-mediated) and non-canonical (caspase-4/5/11-mediated) pyroptosis pathways. To investigate pyroptosis induced by H6N6 virus infection at the protein level, M1 macrophages were pre-treated with inhibitors (MCC950, AC-YVAD-CMK, LDC7559) and then infected with the virus. WB was used to detect GSDMD-N and GSDMD protein expression in each infection group. Compared with the mock group, GSDMD-N protein levels decreased in each infection group at 12 hpi after treatment with the three inhibitors (*p* < 0.001) (Figure 5G–I). This reduction in GSDMD-N protein levels at the protein level confirms that H6N6 infection induces pyroptosis in M1 macrophages. Following pyroptosis, LDH, IL-1β, and IL-18 were released extracellularly through plasma membrane pores formed by GSDMD-N. Post-treatment with inhibitors (MCC950, AC-YVAD-CMK, LDC7559), the release of LDH from infected M1 macrophages was significantly reduced (*p* < 0.05) (Figure 5J,M), and the release of IL-1β was reduced at 24 hpi (*p* < 0.05) (Figure 5K,N). Post-treatment with the NLRP3 inhibitor (MCC950) and caspase-1 inhibitor (AC-YVAD-CMK) decreased IL-18 release from infected M1 macrophages at 12 and 24 hpi (*p* < 0.05). Treatment with the GSDMD inhibitor (LDC7559) specifically reduced IL-18 release at 24 hpi (*p* < 0.05) (Figure 5L,O).

## 4. Discussion

The frequent cross-species transmission of avian influenza virus poses a significant threat to human health. In recent years, the widespread circulation of H6 AIV in poultry populations, its expanding host range to mammals, and its potential implications for human infection have garnered substantial concern [29]. The predominance of H6N6 among avian influenza strains raises public health concerns due to its frequent isolation from poultry and wild birds, capacity to reassort with other subtypes (e.g., H5, H7, H9), and potential to generate novel viruses with cross-species infectivity [30,31]. Furthermore, emerging receptor-binding features, such as hemagglutinin mutations in the receptor binding domain (e.g., Q226L/G228S), may alter pandemic potential by enabling binding to human-like SAα-2,6 Gal receptors, facilitating efficient human infection and increasing the risk of human-to-human transmission [6]. This study confirms that LPAIV H6N6 can effectively replicate in and infect alveolar macrophages and their M1-polarized macrophages. Moreover, it induces pyroptosis in human M1 macrophages by activating the NLRP3/Caspase-1/GSDMD pathway, revealing the inherent pro-inflammatory risk associated with H6 virus infection. This provides novel mechanistic insights into the molecular basis of AIV-induced pulmonary injury and identifies promising therapeutic targets for future antiviral strategies.

AIVs primarily infected human lower respiratory tract cells, including type II alveolar epithelial cells and macrophages. This infection caused pulmonary tissue damage, inflammatory responses, and might lead to respiratory dysfunction or failure [7,8,23,25]. Furthermore, AIV infection might hyperactivate the NLRP3 inflammasome, triggering a cytokine storm that led to severe acute lung injury [32,33]. Both human influenza viruses and the high-pathogenic avian influenza virus (HPAIV) H7N9 virus induced pyroptosis through activation of intracellular signaling pathways [34]. Specifically, HPAIV H7N9 infection induces alveolar cells pyroptosis through the caspase-3/GSDME pathway, which leads to lethal cytokine storms [23]. Lung tissue macrophages are widely distributed throughout the lung and are the most abundant immune cell population in the lungs. They play a key role in immune responses and participate in almost all physiological and pathological processes of the lungs [35,36,37,38]. Our study demonstrates that LPAIV H6N6 infection of M1 macrophages can upregulate NLRP3, caspase-1, and GSDMD mRNA. Activated caspase-1 can cleave IL-1β and IL-18 precursor, promoting IL-1β and IL-18 release. In addition, caspase-1 also cleaves GSDMD to generate GSDMD-N, which forms pores in the cell membrane, ultimately leading to cell swelling, lysis and death. Thus, LPAIV H6N6 infection induces M1 macrophage pyroptosis via the NLRP3/Caspase-1/GSDMD pathway. However, the pathways by which H6N6 and H7N9 viruses induced cell pyroptosis were different. This discrepancy may stem from two factors: (1) divergent pathogenicity—H7N9 is a highly pathogenic avian influenza virus, whereas H6N6 is of low pathogenicity; (2) distinct target cells, as H7N9 primarily infects alveolar cells, while H6N6 preferentially targets M1 macrophages in our study. This suggests that AIV-induced pyroptosis pathways are influenced by several factors, including viral replication fitness, pathogenicity, and host cell tropism. In addition to these viral and cell-type factors, the host’s genetic background, particularly polymorphisms in inflammasome-related genes such as NLRP3, may play a significant role in determining pyroptosis pathway activation. This genetic variability could contribute to differences in disease severity among individuals infected with AIV and warrants further investigation. Meanwhile, our findings changed the previous understanding that only HPAIV (such as H7N9) induce pyroptosis—demonstrating that LPAIV can also trigger this programmed cell death. H6N6 virus infection induces pyroptosis in M1 macrophages, leading to a large release of IL-1β/IL-18, which may synergize with the inherent pro-inflammatory characteristics of M1, cascading and amplifying the inflammatory response. This dual action may explain the potential mechanism by which LPAIV can still cause severe lung injury, for example, some cases of LPAIV H10 infection in humans can cause severe lung damage and even death [39,40,41,42], suggesting that immunopathologic damage may be more critical than direct viral damage. This highlights the need to consider both antiviral and immunomodulatory strategies to address LPAIV-induced lung injury, rather than focusing solely on inhibiting viral replication. Our findings provide a mechanistic basis for the potential “cytokine storm” that could occur in future H6N6 infection humans.

Based on the aforementioned study confirming that H6N6 virus infection induces pyroptosis in M1 macrophages, this study further confirms that this process is mediated through the NLRP3-CASP1-GSDMD pathway. Although Influenza A viruses activate NLRP3 through multiple pathways, the specific mechanisms remain incompletely defined. The NLRP3 inflammasome is an upstream activating factor that triggers pyroptosis. Pharmacological blockade of caspase-1 or genetic knockdown of the NLRP3 pathway can suppress disease pathogenesis and mitigate progression. Although GSDMD-mediated pyroptosis protects against pathogens’ infections [43,44], aberrant activation of GSDMD can trigger detrimental inflammatory cascades, such as cell lysis and the sustained release of inflammatory cytokines. As a key upstream regulator of cytokine storms, inhibiting GSDMD cleavage effectively dampens host inflammatory responses. MCC950 is a selective small molecule inhibitor that specifically inhibits the activation of NLRP3 but does not affect AIM2, NLRC4, or NLRP1. AC-YVAD-CMK is a selective irreversible Caspase-1 inhibitor that can block IL-1β and IL-18 maturation and effectively attenuate inflammation. LDC7559 is a GSDMD-specific inhibitor that directly targets GSDMD-N, effectively reducing IL-1β release and thereby suppressing inflammation. In this study, M1 macrophages were pre-treated with the NLRP3 inhibitor (MCC950), the Caspase-1 inhibitor (AC-YVAD-CMK), and the GSDMD inhibitor (LDC7559), followed by inoculation of the cells with H6N6 virus to detect the expression of key factors in the NLRP3/Caspase-1/GSDMD pathway. The specific inhibitors (MCC950, AC-YVAD-CMK, LDC7559) significantly inhibited pyroptosis markers (NLRP3, Caspase-1, GSDMD-N, IL-1β/IL-18) and cell lysis, further confirming that H6N6 virus infection induces pyroptosis in M1 macrophages via the NLRP3/CASP1/GSDMD pathway. Notably, the GSDMD inhibitor LDC7559 directly blocks membrane pore formation, offering a novel therapeutic strategy that targets the pyroptosis executioner protein for AIV infection. This study characterizes the pyroptosis pathway activated by the H6N6 strain A/CK/DG/2953/2021. However, it is essential to recognize the considerable heterogeneity in pathogenicity and receptor binding affinity among circulating H6N6 strains. Extensive surveillance reveals a spectrum of virulence in mammalian models, ranging from strains with avirulent phenotypes to those causing fulminant infection and 100% mortality in mice [45]. Similarly, most H6N6 viruses retain avian-like receptor binding (SAα-2,3-Gal). However, emergent variants with mutations like L226 and S228 in hemagglutinin (H3 numbering) show increased affinity for human-like receptors (SAα-2,6-Gal), raising their zoonotic potential [45]. This genotypic and phenotypic diversity, driven by dynamic reassortment, highlights the non-uniform risk posed by H6N6 viruses and the need for continuous surveillance of their evolution.

The limitations of this study. First, our findings primarily demonstrate that H6N6 virus infection (MOI = 0.1) induces pyroptosis in M1 macrophages in vitro. Future studies that incorporate a broader range of MOIs and more frequent time points will be critical to fully elucidate the kinetic and dose-dependent mechanisms underlying H6N6-induced pyroptosis and cytokine release in macrophages. We should also explore the relationship between pyroptosis and pulmonary injury by dynamically correlating pyroptosis markers with histopathological indices, lung inflammation scores, and alveolar damage severity. Second, the morphological evidence of pyroptosis from SEM (Figure 3C) is only qualitative: constraints of our current experimental setup (e.g., maximum achievable magnification, limited image sets) led to insufficient observable membrane pores (a pyroptosis hallmark) for reliable quantification of parameters like pore size or frequency, and future work will optimize sample preparation and increase imaging replicates to address this. Third, the molecular mechanism through which H6N6 activates NLRP3 remains unclear. Therefore, further research is needed to clarify how influenza viruses activate upstream pathways that trigger NLRP3 inflammasome assembly. Finally, this study focused solely on the canonical pyroptosis pathway and its key components, such as NLRP3. Future research should explore alternative pyroptotic pathways, including the non-canonical caspase-4/11-mediated and caspase-3/GSDME pathways. Additionally, single-cell RNA sequencing should be employed to analyze heterogeneity among macrophage subpopulations in their pyroptotic responses.

Our findings robustly delineate the canonical NLRP3/caspase-1/GSDMD pathway as the primary mechanism driving pyroptosis in H6N6-infected human M1 macrophages. However, emerging evidence suggests that alternative pathways may be activated in a cell-type-dependent manner. For instance, the caspase-3/GSDME pathway has been well-documented in alveolar epithelial cells during infections with other influenza subtypes including H1N1, H9N2, and H10N3 [46,47,48]. In contrast, the role of the non-canonical caspase-4/5 pathway in influenza pathogenesis remains largely unexplored, presenting an important area for future investigation. While our study did not directly assay these alternatives in M1 macrophages (where canonical signaling predominates), we acknowledge their potential relevance for H6N6 pathogenesis across heterogeneous lung cell populations. This underscores the need for future work to systematically map cell death pathways activated by H6N6 in diverse human lung cells (e.g., alveolar epithelial cells, endothelial cells) using cell-type-specific models and multi-omics approaches. Such efforts will elucidate the full spectrum of immunopathological mechanisms and inform targeted therapeutic strategies against H6N6-induced lung injury. Furthermore, elucidating how H6N6 infection triggers NLRP3 inflammasome activation through potential viral proteins (e.g., PB1-F2, NS1) or host-derived signals (e.g., mitochondrial ROS, potassium efflux) remains essential for understanding the initial events in pyroptotic cascade activation. Elucidating these initial activation events will be essential for constructing a complete molecular pathogenesis model of H6N6-induced inflammatory cell death.

## 5. Conclusions

This study demonstrates that LPAIV H6N6 can efficiently replicate in and infect human alveolar macrophages and M1-polarized macrophages. Crucially, H6N6 infection in M1 macrophages induces pyroptosis via the NLRP3/Caspase-1/GSDMD pathway, which may exacerbate pro-inflammatory responses. Given that M1 macrophages naturally produce pro-inflammatory cytokines, concurrent pyroptosis—releasing substantial IL-1β and IL-18—may exacerbate pro-inflammatory responses. This cascade may trigger a pulmonary cytokine storm, which could be a mechanism underlying H6N6 virus pathogenesis. Collectively, these findings offer new approaches to understand the pathogenic mechanisms of LPAIVs and to identify promising therapeutic targets against AIV-induced immunopathology.

## Data Availability

The original contributions presented in the study are included in the article; further inquiries can be directed to the corresponding authors.

## Abbreviations

The following abbreviations are utilized throughout this manuscript:

AIV	avian influenza virus
BSA	bovine serum albumin
CARD	caspase activation and recruitment domain
GSDMD	Gasdermin D
HPAIV	high-pathogenic avian influenza virus
hpi	hours post-infection
IHC	immunohistochemistry
LDH	Lactate Dehydrogenase
LPAIV	low-pathogenic avian influenza virus
LPS	lipopolysaccharides
MCSF	macrophage colony-stimulating facto
MDCK	Madin-Darby Canine Kidney
MOI	multiplicities of infection
NP	nucleoprotein
OD	optical density
PAMPs	pathogen-associated molecular patterns
PCD	programmed cell death
PMA	phorbol 12-myristate 13-acetate
SEM	Scanning Electron Microscopy
TCID_50_	50% tissue culture infectious dose
WB	western blot
WHO	World Health Organization
